# Vascularized composite allograft deceased donation in the United States

**DOI:** 10.3389/frtra.2024.1520970

**Published:** 2025-01-07

**Authors:** Wida S. Cherikh, Samantha M. Noreen, Alexandra Lewis, Sarah E. Booker, Jesse Howell, Erin M. Schnellinger, Jennifer L. Wainright, Christopher C. Curran

**Affiliations:** ^1^ Research Department, United Network for Organ Sharing, Richmond, VA, United States; ^2^ Organ Donation Services, New England Donor Services, Waltham, MA, United States

**Keywords:** vascularized composite allograft (VCA), deceased donor, organ yield, observed-to-expected organ yield, uterus

## Abstract

Vascularized composite allograft (VCA) transplantation represents a significant advancement in reconstructive surgery and offers hope to individuals who experienced congenital disorders or severe tissue injuries to restore physical appearance, function, and enhance quality of life. VCA recovery introduces complexities to conventional solid organ recovery, and there remain concerns regarding the potential impact of VCA recovery on non-VCA organs for transplant. The current retrospective study examines deceased donor characteristics and observed-to-expected (O/E) organ yield ratios for 51 VCA donors recovered in the US between July 4, 2014 and March 31, 2024, compared with a contemporary cohort of non-VCA donors recovered in 2023. Among the VCA donors, 17 donated a uterus, 15 each donated head and neck and upper limbs, 4 were abdominal wall donors, and 2 donated external male genitalia. The findings indicate that VCA donors tended to be younger (18–34 years old), predominantly White, non-Hispanic, and had no history of diabetes, along with lower weight, lower kidney donor profile index, and lower ejection fraction. The analysis revealed that VCA donors had higher observed overall organ yield than expected (O/E: 1.24, 95% CI: 1.16–1.33), with better-than-expected organ yields across VCA types. The number of deceased VCA donors in the US is still relatively small compared to the overall donor population. As the field continues to evolve and more data becomes available, further analyses need to be conducted to understand the demographics of VCA donors and the potential impact of VCA donation within the donation and transplant system.

## 1 Introduction

The field of transplantation has seen remarkable advancements in the last few decades, especially in vascularized composite allografts (VCA). This evolving and complex field of transplantation provides unique opportunities for individuals with severe tissue damage who experienced trauma, infection, or had congenital conditions to regain function and enhance quality of life. VCA transplantation involves transplanting multiple structures that may include organs such as uterus, connective tissues, skin, bone, blood vessels, and nerves.

The first successful VCA transplant in the US was a laryngeal transplant in 1998 (1) followed by an upper limb transplant in 1999 (2). Since these initial successes, VCA transplantation in the US has expanded to include face, scalp, trachea, abdominal wall, penis, and uterus (3–5). On July 3, 2014, the Organ Procurement and Transplantation Network (OPTN) was granted oversight of VCA procurement and transplantation in the United States (6).

With these advances, it is imperative that the recovery of VCA grafts from deceased donors does not inhibit the recovery of other solid organs for transplant. VCA recovery adds a variable amount of time to the donor recovery process depending on factors including the type of VCA and the surgical techniques used and requires careful coordination with the abdominal and thoracic organ recovery teams (7). The VCA and organ donation and transplantation communities are mindful of this, and several programs have published their processes and recommendations for recovering VCAs alongside solid organ recoveries (8–13). In 2018, the OPTN VCA Transplantation Committee produced a guidance document on optimizing VCA recovery from deceased donors, which included recommendations for coordinating VCA recovery with solid organ recovery (14). In 2023, the Committee updated this guidance to reflect the evolution of the field since 2018 (7).

An analysis done by Vece et al. (2020) found that solid organ yield from VCA donors recovered during 2008–2017 was as good or better than expected, suggesting that VCA procurement can be done successfully without detriment to solid organ recovery and transplantation. There have been notable advancements in VCA transplantation in the US since 2017, including the rapid expansion of uterus transplantation following the first uterus transplants in 2016, the first successful combined upper limb and face transplant, and the first trachea transplant (5). At the same time, organ procurement organizations (OPOs) have faced increasing pressure to recover more solid organs for transplant, without Centers for Medicare and Medicaid Services (CMS) counting VCA grafts towards OPO performance measures (15), which could disincentivize allocation and recovery of VCA organs for fear that it could put the recovery and transplant of other organs at risk.

Considering these changes in the field of VCA transplantation and in the broader donation and transplantation community, we sought to examine recent trends in VCA donation in the US and solid organ yield for a contemporary cohort of deceased VCA donors. Understanding changes in these trends is particularly relevant given the expansion of uterus transplantation and landscape of types of VCA transplant being practiced. We assessed deceased donor characteristics and observed-to-expected organ yield ratios for VCA donors recovered since the OPTN was granted oversight, compared with a contemporary cohort of non-VCA donors recovered in 2023. We hypothesized that solid organ transplantation from VCA donors would continue to be as good or better than expected due to advancemets in the field, despite challenges.

## 2 Methods

### 2.1 Cohort

This analysis included VCA deceased donors who donated between July 3, 2014 and March 31, 2024. All VCA donors in the cohort had a VCA organ transplanted and at least one other solid organ recovered for transplant. All VCA donors during this time also had a solid organ recovered. For comparison purposes, we included a recent cohort of deceased donors with at least one solid organ and no VCA organs transplanted who donated between January 1, 2023 and December 31, 2023.

### 2.2 Data source

This retrospective study used the OPTN data as of July 5, 2024. The OPTN data system includes data on all donors, wait-listed candidates, and transplant recipients in the US, submitted by the members of the OPTN, and has been described elsewhere (16). The Health Resources and Services Administration (HRSA), US Department of Health and Human Services provides oversight to the activities of the OPTN contractor.

The current study used patient-level, non-identifiable data extracted from the OPTN research database and was determined to be IRB-exempt by the Chesapeake Institutional Review Board.

### 2.3 Statistical analysis

We summarized the demographic and basic clinical characteristics of deceased donors, comparing those with transplanted VCA organs to those without. We presented the distribution of donor characteristics using counts and percentages for categorical variables, and medians with interquartile ranges (IQR) for continuous variables. Missing values were enumerated but not included in statistical testing. Characteristics were compared between VCA and non-VCA donors using chi-square tests for categorical variables and Wilcoxon rank-sum tests for continuous variables. Additionally, we stratified characteristics of VCA donors by VCA type, including abdominal wall, external male genitalia, head and neck, upper limb, and uterus. Head and neck transplants included face, scalp, trachea, and larynx; upper limb transplants included both unilateral and bilateral upper limb.

To compare whether the number of organs transplanted was above or below expectation for VCA donors relative to deceased donors without VCA organs transplanted, we used observed-to-expected (O/E) yield ratios. We calculated the expected organ yield by applying the Scientific Registry of Transplant Recipients (SRTR) risk-adjusted deceased donor yield model coefficients released in January 2024 (17). Each solid organ (heart, intestine, kidney, liver, lung, pancreas) has a separate risk-adjustment model, developed on donors from whom at least one organ was recovered for the purpose of transplant. We obtained the expected number of organs transplanted for all organs (aggregated), as well as for kidney, liver, heart, lung, and pancreas for VCA and non-VCA donors, respectively. The observed organ yield is the actual number of transplanted solid organs (heart, intestine, kidney, liver, lung, pancreas, and aggregated). Each O/E ratio was then calculated by dividing the observed number of transplanted organs and the expected number of transplanted organs. We assessed O/E ratios overall and by solid organ type for VCA and non-VCA donors, as well as for each VCA type for VCA deceased donors.

We applied bootstrapping techniques (18, 19) to calculate 95% confidence intervals around the O/E ratios and to evaluate statistical significance. A total of 1,000 bootstrapped samples were generated under the null hypothesis that the O/E ratio was 1.0 (indicating that the observed yield was equal to the expected yield).

A sensitivity analysis was conducted using the January 2019 SRTR models, as the cohort of VCA donors spanned a longer period of time. Only the O/E results using the January 2024 models are discussed as the results were similar.

All analyses were conducted using R version 4.3.3 (20).

## 3 Results

### 3.1 Donor characteristics

Between July 3, 2014, and March 31, 2024, there were 51 deceased donors with VCA organs transplanted. Two of these VCA donors each donated two VCA organs (face and upper limb); one donor donated to two different recipients, while the other donated both organs to the same recipient. These donors were counted twice when describing deceased VCA donors by VCA type, and only once when comparing deceased VCA and non-VCA donors. These donors were counted once under the head and neck VCA type for O/E ratio results.

There were 4 abdominal wall, 2 external male genitalia, 15 head and neck, 15 upper limb, and 17 uterus deceased donors. These donors were recovered at 17 OPOs, representing 30% of the 56 OPOs in the US as of March 2024 and 9 of the 11 OPTN regions.

The donations from these 51 donors resulted in 53 deceased donor VCA transplants and 255 transplanted non-VCA organs. The non-VCA organ transplanted included 34 hearts, 6 intestines, 93 kidneys, 51 livers, 53 lungs, and 18 pancreata.

Compared with non-VCA donors, VCA donors were more likely to be younger, have lower kidney donor profile index (KDPI), have better lung function, and have a circumstance of death of “Other” (Table 1). The majority (63%) of VCA donors were 18–34 years old as compared to 25% of non-VCA donors (*p* < 0.001), with only one VCA donor older than 50. There were more female VCA donors than non-VCA donors, primarily driven by female uterus donors; 17 of the 26 female donors were uterus donors. After excluding uterus donors, only 9 (27%) were female, which was a much lower proportion than that of non-VCA donors (38%).

**Table 1:** Characteristics of deceased VCA donors between July 3, 2014 and March 31, 2024 and deceased non-VCA donors in 2023.

Characteristic	VCA donor	Non-VCA donor	*p*-value*
	*N* = 51	*N* = 14,330	
Donor age			<0.001
<18	4 (7.84%)	849 (5.92%)	
18–34	32 (62.7%)	3,615 (25.2%)	
35–49	14 (27.5%)	4,529 (31.6%)	
50–64	1 (1.96%)	4,252 (29.7%)	
65+	0 (0%)	1,085 (7.57%)	
Donor birth sex			0.071
Male	25 (49.0%)	8,927 (62.3%)	
Female	26 (51.0%)	5,403 (37.7%)	
Donor race/Ethnicity			0.360
White, non-Hispanic	39 (76.5%)	9,255 (64.6%)	
Black, non-Hispanic	7 (13.7%)	2,343 (16.4%)	
Hispanic	5 (9.80%)	2,123 (14.8%)	
Asian, non-Hispanic	0 (0%)	361 (2.52%)	
Other, non-Hispanic	0 (0%)	248 (1.73%)	
Donor blood type			0.240
A	14 (27.5%)	5,201 (36.3%)	
AB	0 (0%)	442 (3.08%)	
B	6 (11.8%)	1,713 (12.0%)	
O	31 (60.8%)	6,974 (48.7%)	
Donor cause of death			0.961
Anoxia	26 (51.0%)	7,089 (49.5%)	
CVA/Stroke	13 (25.5%)	3,319 (23.2%)	
CNS tumor	0 (0%)	49 (0.34%)	
Head trauma	10 (19.6%)	3,337 (23.3%)	
Other	2 (3.92%)	536 (3.74%)	
Donor mechanism of death			0.103
Asphyxiation	5 (9.80%)	684 (4.77%)	
Blunt injury	6 (11.8%)	2,407 (16.8%)	
Cardiovascular	5 (9.80%)	2,835 (19.8%)	
Drug intoxication	13 (25.5%)	2,581 (18.0%)	
Gunshot wound	6 (11.8%)	961 (6.71%)	
Intracranial hemorrhage/Stroke	13 (25.5%)	3,384 (23.6%)	
Other	3 (5.88%)	1,478 (10.3%)	
Donor circumstance of death			0.049
Accident, non-MVA	11 (25.6%)	2,933 (23.1%)	
Death from natural causes	15 (34.9%)	6,523 (51.3%)	
MVA	6 (14.0%)	1,638 (12.9%)	
Other	11 (25.6%)	1,630 (12.8%)	
Missing	8	1,606	
KDPI			<0.001
0%–20%	33 (64.7%)	3,089 (21.6%)	
21%–34%	6 (11.8%)	2,125 (14.8%)	
35%–85%	11 (21.6%)	7,218 (50.4%)	
86%–100%	1 (1.96%)	1,898 (13.2%)	
PHS risk factors			0.113
No	46 (92.0%)	11,564 (82.5%)	
Yes	4 (8.00%)	2,460 (17.5%)	
Missing	1	306	
DCD donor			<0.001
No	51 (100.0%)	9,808 (68.4%)	
Yes	0 (0%)	4,522 (31.6%)	
Donor hepatitis C status			0.116
Negative	50 (98.0%)	12,989 (90.6%)	
Positive	1 (1.96%)	1,341 (9.36%)	
Donor HIV status			1.000
Negative	48 (100.0%)	14,290 (99.8%)	
Positive	0 (0%)	34 (0.24%)	
Missing	3	6	
Donor history of cancer			0.798
No	49 (98.0%)	13,561 (96.3%)	
Yes	1 (2.00%)	519 (3.69%)	
Missing	1	250	
Cardiac arrest after brain death			1.000
No	48 (94.1%)	9,296 (94.7%)	
Yes	3 (5.88%)	525 (5.35%)	
Missing	0	4,509	
Clinical infection: blood			0.460
No	43 (89.6%)	12,125 (84.7%)	
Yes	5 (10.4%)	2,191 (15.3%)	
Missing	3	14	
Clinical infection: lung			0.025
No	8 (16.0%)	4,545 (31.7%)	
Yes	42 (84.0%)	9,771 (68.3%)	
Missing	1	14	
Clinical infection: urine			0.169
No	37 (77.1%)	12,196 (85.2%)	
Yes	11 (22.9%)	2,120 (14.8%)	
Missing	3	14	
Clinical infection: other			0.592
No	46 (93.9%)	12,973 (90.6%)	
Yes	3 (6.12%)	1,343 (9.38%)	
Missing	2	14	
Current cigarette use			1.000
No	1 (14.3%)	502 (16.1%)	
Yes	6 (85.7%)	2,622 (83.9%)	
Missing	44	11,206	
History of cigarette use			0.207
No	42 (85.7%)	10,602 (77.1%)	
Yes	7 (14.3%)	3,149 (22.9%)	
Missing	2	579	
Current cocaine use			1.000
No	4 (36.4%)	651 (34.3%)	
Yes	7 (63.6%)	1,247 (65.7%)	
Missing	40	12,432	
History of cocaine use			0.928
No	36 (76.6%)	6,604 (75.0%)	
Yes	11 (23.4%)	2,207 (25.0%)	
Missing	4	5,519	
Current other drug use			1.000
No	3 (10.7%)	547 (12.4%)	
Yes	25 (89.3%)	3,849 (87.6%)	
Missing	23	9,934	
History of other drug use			0.607
No	20 (41.7%)	4,131 (46.4%)	
Yes	28 (58.3%)	4,767 (53.6%)	
Missing	3	5,432	
Ejection fraction (%)			0.037
Median (IQR)	56 (55, 63)	60 (55, 65)	
Missing	2	3,977	
Heavy alcohol use			0.073
No	43 (87.8%)	10,374 (75.7%)	
Yes	6 (12.2%)	3,322 (24.3%)	
Missing	2	634	
Donor height (cm)			0.826
Median (IQR)	170 (164, 180)	170 (163, 178)	
Missing	0	1	
History of diabetes and insulin dependence			0.038
Diabetes with insulin dependence	0 (0%)	787 (5.59%)	
Diabetes with no insulin dependence	0 (0%)	931 (6.62%)	
Diabetes with unknown insulin dependence	0 (0%)	317 (2.25%)	
No diabetes	50 (100.0%)	12,034 (85.5%)	
Missing	1	261	
History of hypertension			0.016
No	40 (80.0%)	8,787 (62.4%)	
Yes	10 (20.0%)	5,287 (37.6%)	
Missing	1	256	
Donor PO_2_			0.007
Median (IQR)	281 (135, 443)	158 (98, 378)	
Missing	0	30	
Donor PO_2_/FiO_2_ ratio			0.054
Median (IQR)	3.69 (1.91, 4.53)	3.00 (1.86, 4.17)	
Missing	0	104	
Previous MI			0.444
No	49 (98.0%)	13,260 (94.5%)	
Yes	1 (2.00%)	767 (5.47%)	
Missing	1	303	
Protein in urine			<0.001
No	31 (60.8%)	4,676 (33.0%)	
Yes	20 (39.2%)	9,478 (67.0%)	
Missing	0	176	
Terminal serum creatinine (mg/dl)			0.107
Median (IQR)	0.95 (0.60, 1.30)	0.99 (0.69, 1.73)	
Missing	0	6	
Donor weight (kg)			0.042
Median (IQR)	78 (67, 84)	82 (68, 98)	
Missing	0	41	

* Distributions of characteristics were compared between VCA and non-VCA donors using Chi-square tests for categorical variables and Wilcoxon rank-sum tests for continuous variables. Missing observations were not included in statistical tests of each characteristic.

Ejection fraction (medians: 56% for VCA donors vs. 60% for non-VCA donors) and donor weight (medians: 78 kg for VCA donors vs. 82 kg for non-VCA donors) were significantly lower for VCA donors compared to non-VCA donors. Compared to non-VCA donors, VCA donors were less likely to have a history of hypertension (*p* = 0.016). The vast majority of both VCA and non-VCA donors were White, non-Hispanic, blood type O, and had anoxia as the cause of death. All VCA donors with known values were brain dead donors, HIV negative, and did not have a history of diabetes.

Characteristics of the 51 deceased VCA donors are further summarized in Table 2 by VCA type (abdominal wall, external male genitalia, head and neck, and upper limb). Overall, the majority of VCA donors were aged 18–34 years old, but a higher percentage of upper limb donors were aged 35–49 compared to any other VCA organ type. After excluding VCA organs specific to a particular sex (uterus and external male genitalia donors), the majority of VCA donors were male.

**Table 2:** Characteristics of deceased VCA donors recovered by VCA type (July 3, 2014 to March 31, 2024).

Characteristic	Abdominal wall	External male genitalia	Head and neck	Upper limb	Uterus
	*N* = 4	*N* = 2	*N* = 15	*N* = 15	*N* = 17
Donor age					
<18	2 (50.0%)	0 (0%)	0 (0%)	2 (13.3%)	0 (0%)
18–34	1 (25.0%)	2 (100.0%)	11 (73.3%)	6 (40.0%)	13 (76.5%)
35–49	1 (25.0%)	0 (0%)	3 (20.0%)	7 (46.7%)	4 (23.5%)
50–64	0 (0%)	0 (0%)	1 (6.67%)	0 (0%)	0 (0%)
65+	0 (0%)	0 (0%)	0 (0%)	0 (0%)	0 (0%)
Donor birth sex					
Male	2 (50.0%)	2 (100.0%)	13 (86.7%)	10 (66.7%)	0 (0%)
Female	2 (50.0%)	0 (0%)	2 (13.3%)	5 (33.3%)	17 (100.0%)
Donor race/Ethnicity					
White, non-Hispanic	2 (50.0%)	2 (100.0%)	12 (80.0%)	14 (93.3%)	11 (64.7%)
Black, non-Hispanic	1 (25.0%)	0 (0%)	1 (6.67%)	1 (6.67%)	4 (23.5%)
Hispanic	1 (25.0%)	0 (0%)	2 (13.3%)	0 (0%)	2 (11.8%)
Asian, non-Hispanic	0 (0%)	0 (0%)	0 (0%)	0 (0%)	0 (0%)
Other, non-Hispanic	0 (0%)	0 (0%)	0 (0%)	0 (0%)	0 (0%)
Donor blood type					
A	1 (25.0%)	0 (0%)	4 (26.7%)	3 (20.0%)	6 (35.3%)
AB	0 (0%)	0 (0%)	0 (0%)	0 (0%)	0 (0%)
B	0 (0%)	2 (100.0%)	0 (0%)	4 (26.7%)	0 (0%)
O	3 (75.0%)	0 (0%)	11 (73.3%)	8 (53.3%)	11 (64.7%)
KDPI					
0%–20%	3 (75.0%)	2 (100.0%)	12 (80.0%)	7 (46.7%)	10 (58.8%)
21%–34%	0 (0%)	0 (0%)	0 (0%)	3 (20.0%)	3 (17.6%)
35%–85%	0 (0%)	0 (0%)	3 (20.0%)	5 (33.3%)	4 (23.5%)
86%–100%	1 (25.0%)	0 (0%)	0 (0%)	0 (0%)	0 (0%)
Current cigarette use					
No	0 (0%)	0 (0%)	0 (0%)	1 (50.0%)	0 (0%)
Yes	0 (0%)	0 (0%)	2 (100.0%)	1 (50.0%)	4 (100.0%)
Missing	4	2	13	13	13
History of cigarette use					
No	3 (100.0%)	2 (100.0%)	12 (85.7%)	13 (86.7%)	13 (76.5%)
Yes	0 (0%)	0 (0%)	2 (14.3%)	2 (13.3%)	4 (23.5%)
Missing	1	0	1	0	0
Current cocaine use					
No	0 (0%)	2 (100.0%)	1 (33.3%)	0 (0%)	1 (33.3%)
Yes	0 (0%)	0 (0%)	2 (66.7%)	3 (100.0%)	2 (66.7%)
Missing	4	0	12	12	14
History of cocaine use					
No	2 (100.0%)	0 (0%)	10 (76.9%)	12 (80.0%)	14 (82.4%)
Yes	0 (0%)	2 (100.0%)	3 (23.1%)	3 (20.0%)	3 (17.6%)
Missing	2	0	2	0	0
Current other drug use					
No	0 (0%)	0 (0%)	2 (25.0%)	1 (14.3%)	0 (0%)
Yes	1 (100.0%)	1 (100.0%)	6 (75.0%)	6 (85.7%)	11 (100.0%)
Missing	3	1	7	8	6
History of other drug use					
No	2 (66.7%)	1 (50.0%)	5 (38.5%)	8 (53.3%)	6 (35.3%)
Yes	1 (33.3%)	1 (50.0%)	8 (61.5%)	7 (46.7%)	11 (64.7%)
Missing	1	0	2	0	0
Donor height (cm)					
Median (IQR)	163 (157, 172)	177 (168, 185)	175 (170, 183)	173 (168, 180)	165 (160, 170)
Donor PO_2_					
Median (IQR)	246 (158, 406)	305 (160, 450)	173 (105, 397)	308 (123, 433)	353 (145, 476)
Donor PO_2_/FiO_2_ ratio					
Median (IQR)	3.89 (2.83, 4.60)	4.54 (4.50, 4.57)	2.88 (2.02, 4.41)	3.40 (1.91, 4.52)	3.79 (1.50, 4.76)
Terminal serum creatinine (mg/dl)					
Median (IQR)	0.87 (0.56, 1.65)	0.67 (0.63, 0.70)	1.10 (0.67, 1.60)	0.95 (0.60, 1.25)	0.90 (0.60, 1.24)
Donor weight (kg)					
Median (IQR)	64 (60, 70)	66 (65, 66)	82 (71, 86)	79 (70, 86)	78 (67, 83)

Head and neck donors tended to have a higher weight, lower pO_2_, lower pO_2_/FiO_2_ ratio, and the highest serum creatinine values compared to the other VCA organ types. Uterus donors had the highest pO_2_ values. There were 4 abdominal wall and 2 external male genitalia donors, so the summary data should be interpreted with caution.

### 3.2 Organ yield

**Figure 1:**
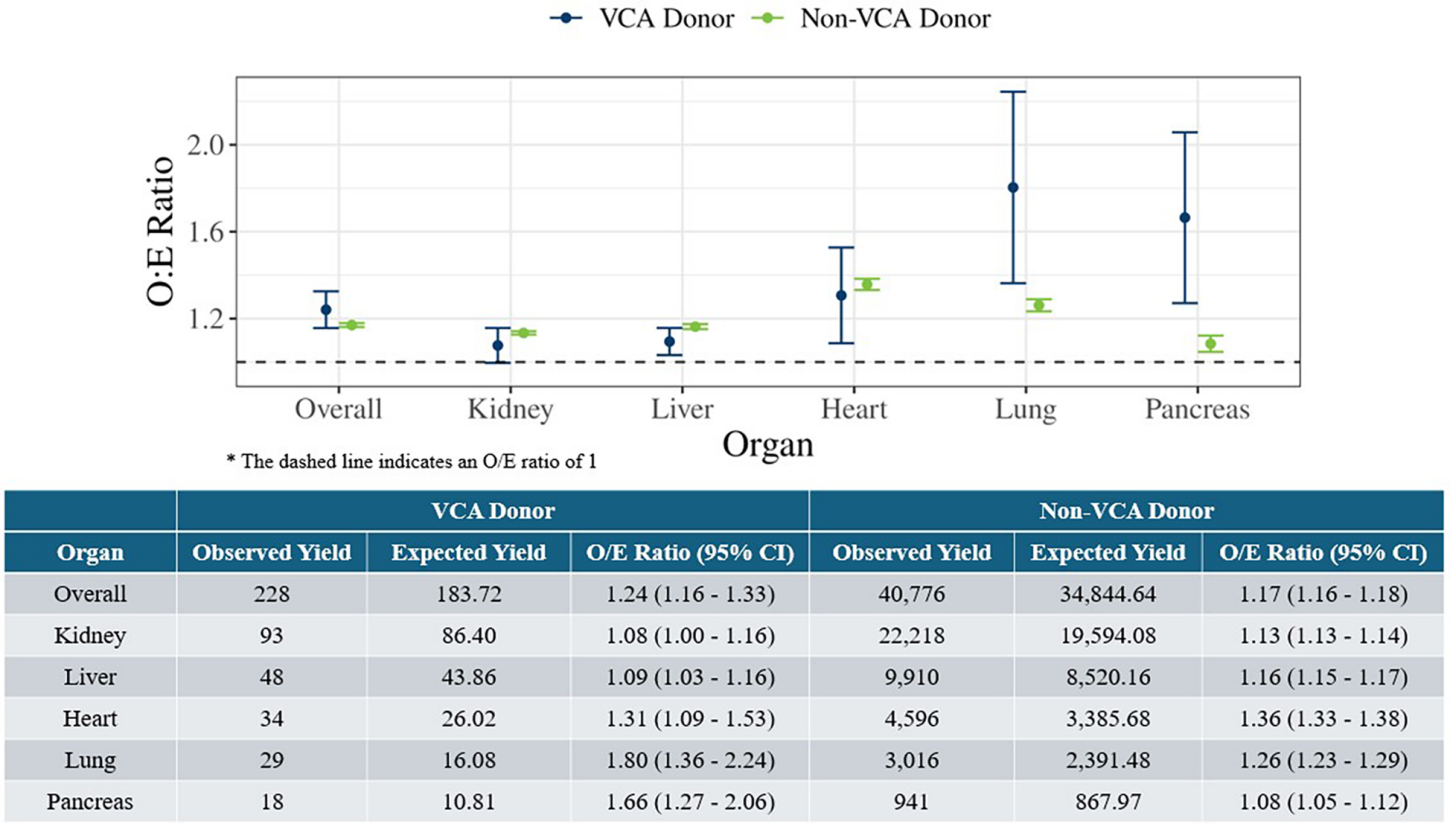
Observed-to-expected organ yield ratios for deceased VCA donors (July 3, 2014 to March 31, 2024) and deceased non-VCA donors in 2023 overall and by solid organ type.

The O/E ratios for deceased donors with at least one organ transplanted were estimated for VCA donors during July 3, 2014 to March 31, 2024 and non-VCA donors in 2023, overall and by solid organ type, as illustrated in Figure 1. The O/E ratios for VCA donors were significantly greater than 1, indicating a higher observed organ yield than expected, i.e., as many or more organs transplanted as expected. The overall O/E ratio for VCA donors was 1.24 (95% CI: 1.16–1.33), which was comparable with the overall O/E ratio for non-VCA donors (1.17, 95% CI: 1.16–1.18). When further analyzed by each solid organ type, the O/E ratios were highest for lung (1.80, 95% CI: 1.36–2.24) and pancreas (1.66, 95% CI: 1.27–2.06), and lowest for kidney (1.08, 95% CI: 1.00–1.16) and liver (1.09, 95% CI: 1.03–1.16). Non-VCA donors also had a higher observed organ yield than expected, both overall and by solid organ type (Figure 1). As indicated by the overlapping confidence intervals, the O/E ratios for VCA and non-VCA donors were not significantly different overall, nor for kidney, liver, and heart.

**Figure 2:**
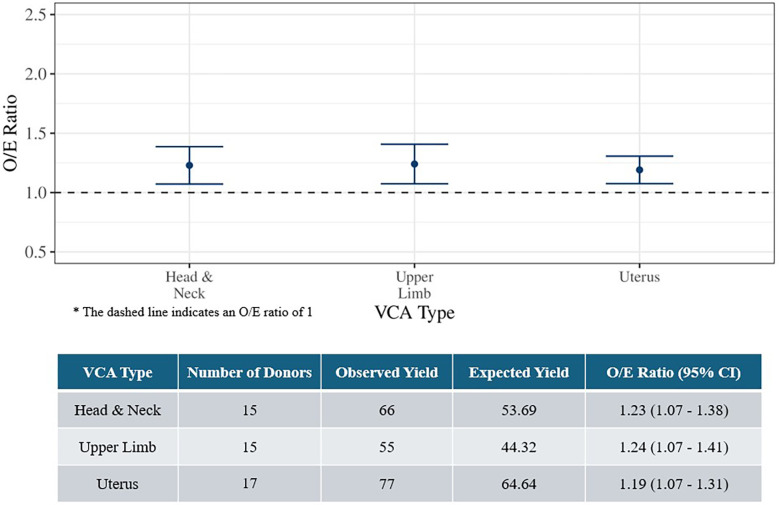
Observed-to-expected organ yield ratios for deceased VCA donors by VCA type (July 3, 2014 to March 31, 2024).

Overall O/E ratios were evaluated by VCA type (Figure 2). Due to small sample sizes, estimates for abdominal wall (*n* = 4) and external male genitalia (*n* = 2) were not calculated. The O/E ratios for head and neck, upper limb, and uterus donors were all significantly greater than 1, again indicating better organ yield than expected.

## 4 Discussion

Deceased VCA donors since 2014 tended to be younger, female, White, non-Hispanic, have a higher incidence of lung clinical infection, and were less likely to have a history of hypertension. While recent trends have shown more marginal deceased organ donors recovered and transplanted in the US to increase organ supply (21), these were not necessarily reflected in contemporary VCA donors. Donor selection for VCA transplants is more conservative due to many recipient and donor factors, including the life-enhancing nature of VCA transplants and the visibility of the graft (3, 22, 23). As a measure of the number of successful organ transplants from donors, observed-to-expected yield ratios indicate that organ yield was as good as or better than expected, based on the national experience. In the current analysis, the utilization of organs from VCA and non-VCA donors was similar, as indicated by a comparison of O/E ratios. It is important to note that in this study we evaluated O/E ratios for a cohort limited to deceased donors with at least one organ transplanted. This aligns with the approach SRTR used to develop the models (19); however, when applied to this cohort, it may result in slightly higher O/E ratios than a cohort that also includes deceased donors with organ(s) recovered and no solid organ transplants. To date, all VCA donations in the U.S. have resulted in transplanted VCA organs.

Compared to VCA donors explored in Wainright et al. (2019) from 1998 to 2017, our contemporary cohort of VCA donors from 2014 to 2024 was more often female, aged 18–34, and had a cause of death of anoxia with mechanism of death of drug intoxication (24). Contemporary VCA donors were also less often White and under 18 years old. Additionally, there was a slight increase in the number of solid organs transplanted from contemporary VCA donors (24, 25). In all instances, VCA donors also donated at least one other solid organ.

Our retrospective study is not without limitations. First, we examined a small number of VCA donors who donated since OPTN was granted VCA oversight, which may have an impact on comparisons with non-VCA donors due to the large imbalance in sample sizes. Consequently, we used data from deceased non-VCA donors in 2023 rather than a longer time period. Additionally, because the sample sizes for calculating O/E ratios for VCA donors overall and by VCA type are small, caution should be exercised when generalizing these results, even after bootstrapping has been applied. We also did not evaluate outcomes of solid organ transplants from these VCA donors, and limited information has been published (26). Future research should consider the post-transplant outcomes for the recipients of these donor organs.

Challenges and opportunities are present for VCA donation and transplantation as the field continues to advance. Current data suggests a relatively limited demand for VCA transplants, with small waitlist sizes. Based on OPTN data there were 14 patients on the VCA waiting list as of October 27, 2024. The small waitlist sizes and limited number of VCA transplant centers in proximity to a small number of OPOs may have resulted in limited need for OPOs to develop procedures to procure VCA organs from donors. Additionally, the requirement for a separate consent for VCA donation (23) adds effort for OPOs to offer VCAs and the exclusion of VCA from the 2020 CMS OPO Final Rule Revisions (15) do not incentivize the allocation and recovery of more VCAs from donors. With alternative therapies for certain VCA types, such as prosthetic limbs for upper and lower limbs, or adoption or surrogacy for women with uterine-factor infertility, benefits of VCA transplant may not outweigh the risks of such surgery (23).

Outside of uterus transplantation, which has progressed rapidly since 2016, one might speculate whether we have reached a “steady state” with VCA transplantation. However, VCA allocation was integrated in the OPTN Computer System, UNet, on September 14, 2023 (27), which may lead to an increase in VCA transplant and donation, potentially changing this trend. OPOs will be better equipped to determine whether any VCA candidates are matched to their donors, and hopefully streamline the VCA offer process, as ineligible candidates are now automatically screened off the match list and potential candidates more easily identified. Previously, this was a manual process done outside of UNet with additional workflow considerations separate from the matching of donors to conventional solid organ candidates.

In conclusion, we examined solid organ utilization from VCA donors in a contemporary cohort and found results consistent with previous publications (24, 25). The diversity of VCA donors remains evident and continues to reflect unique aspects of VCA recipients, particularly in terms of physical matches in appearance and ability to select donors with lowest risk for recipients (23). With the growth in newer types of VCA transplantation, there has been a notable shift particularly toward a larger proportion of female VCA donors, which is reflective of the increase in uterus transplantation.

While VCA donation is still limited in the U.S. and there are additional challenges to increasing overall organ donation, our findings suggest that VCA donation does not negatively impact solid organ donor yields. Understanding the demographics of VCA donors and the potential impact of VCA donation to the donation of other organs is essential for enhancing donor procurement strategies and ultimately increasing the availability of these life-changing gifts.

## Data Availability

The datasets presented in this article are not readily available because the use of OPTN data is governed by the Data Use Agreement. Each researcher must submit their own request to obtain the datasets used in the current analysis. Requests to access these datasets should be submitted at the following link: https://optn.transplant.hrsa.gov/data/view-data-reports/request-data/.
